# Crystal structure of bis­[μ-2-(diiso­propyl­phosphor­yl)propan-2-olato-κ^3^ *O* ^1^,*O* ^2^:*O* ^1^]bis­[chlorido­oxidovanadium(IV)]

**DOI:** 10.1107/S2056989016007362

**Published:** 2016-05-06

**Authors:** Mathias Glatz, Berthold Stöger, Matthias Weil, Karl Kirchner

**Affiliations:** a Institute of Applied Synthetic Chemistry, Vienna University of Technology, Getreidemarkt 9/163-OC, 1060 Vienna, Austria; b Institute of Chemical Technologies and Analytics, Vienna University of Technology, Getreidemarkt 9, A-1060 Vienna, Austria

**Keywords:** crystal structure, binuclear centrosymmetric complex, vanadium, square-pyramidal coordination geometry

## Abstract

The dinuclear mol­ecule of the title complex, [VOCl{μ-OC(Me)_2_P(iPr)_2_-κ^2^ *O*}]_2_ or [V_2_(C_9_H_20_O_2_P)_2_Cl_2_O_2_], which was obtained due to an unexpected oxidation reaction, is centrosymmetric, with the inversion centre located in the middle of the central V_2_O_2_ core. These core O atoms arise from the symmetry-related 2-(diiso­propyl­phosphor­yl)propan-2-olate dianions. The V^IV^ atom is additionally bonded to one terminal Cl ligand, the second O atom of the dianion and double bonded to a vanadyl O atom, leading to an overall distorted square-pyramidal VO_4_Cl coordination polyhedron with the vanadyl O atom as the apex. An intra­molecular C—H⋯Cl contact helps to establish the mol­ecular configuration. In the crystal, mol­ecules are stacked in rows parallel to [001] and are linked by C—H⋯Cl contacts to form chains running in the same direction.

## Abstract 2

The dinuclear title mol­ecular complex is centrosymmetric, with the V^IV^ atom in a distorted square-pyramidal coordination environment.

## Chemical context   

**Figure 1:**
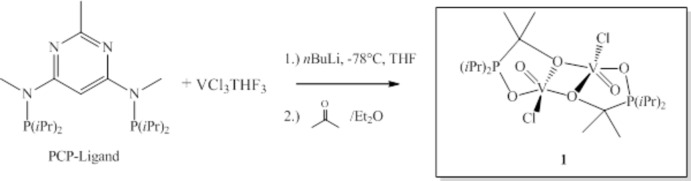
Schematic representation of the attempted formation of a vanadium(III) complex with the PCP ligand.

Tridentate pincer ligands play an important role in coordination chemistry and have found various applications, for example in the fields of catalysis, synthetic chemistry or mol­ecular recognition (Szabo & Wendt, 2014 ▸). Whereas a plethora of second- and third-row transition metal complexes with pincer ligands of various types (*e.g*. PNP- or PCP-coordinating) has been reported in recent years, investigations with respect to first-row transition metals are scarce (Murugesan & Kirchner, 2016 ▸). During a current project to prepare the first vanadium pincer complexes (Mastalir *et al.*, 2016 ▸), we also attempted to synthesize a vanadium(III) PCP-complex according to the reaction scheme presented in Fig. 1 ▸. However, during the course of crystallization using a diffusion method in the presence of traces of water and/or oxygen, a variety of side-reactions took place. Those included oxidation of vanadium(III) to vanadium(IV) and of phospho­rus, cleavage of the P—N bond and the formation of a P—C bond. As a result, the vanadium(IV) title complex [VOCl{μ-OC(Me)_2_P(iPr)_2_-κ^2^ *O*}]_2_, (1), was obtained instead. Its crystal structure is reported in this communication.
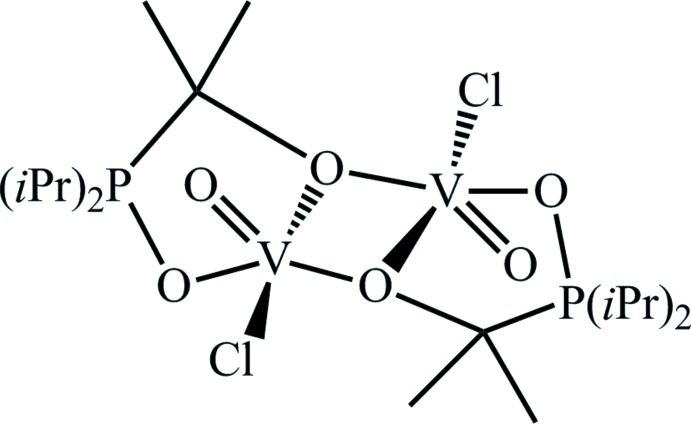


## Structural commentary   

**Figure 2:**
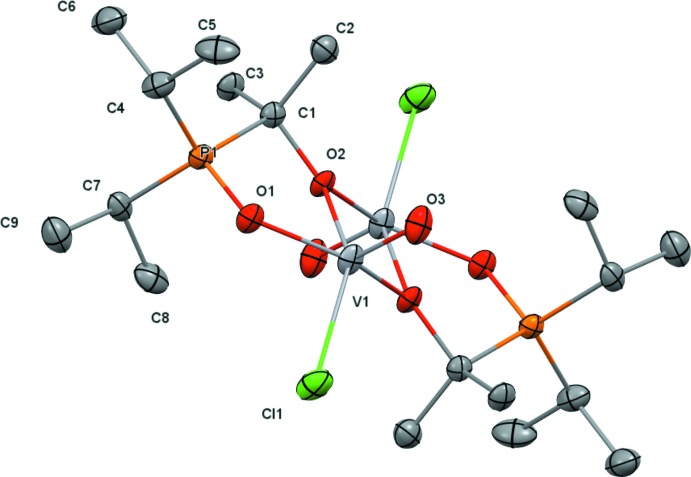
The mol­ecular structure of the binuclear complex with displacement ellipsoids drawn at the 50% probability level. Unlabelled atoms are generated by symmetry code (−*x* + 1, −*y*, −*z* + 1).

The dinuclear mol­ecular complex of (1) is centrosymmetric, containing a rhombic V_2_O_2_ core [V—O—V angle 105.36 (8)°, O—V—O angle 74.64 (7)°]. The V^IV^ atom adopts a distorted square-pyramidal geometry with atoms O1, O2, O2^i^ and Cl1 forming the basal plane and vanadyl atom O3 the apex [for symmetry operator (i), see Fig. 2 ▸]. The V^IV^ atom is displaced by 0.6157 (5) Å from the least-squares plane towards the apex. The Addison τ-parameter (Addison *et al.*, 1984 ▸), as calculated by −0.01667·(139.45)+0.01667·(148.82) = 0.156, also points to this coordination (a value of 0 refers to an ideal square-pyramidal, a value of 1 to an ideal trigonal-bipyramidal coordination). The V=O double-bond length of 1.586 (2) Å is in the typical range of those reported in similar dimeric oxido-chlorido-vanadium(IV) complexes containing alkoxide bridges (Cui *et al.*, 2010 ▸; Crans *et al.*, 1991 ▸; Foulon *et al.*, 1993 ▸; Janas *et al.*, 1997 ▸; Rosenthal, 2009 ▸).

## Supra­molecular features   

**Figure 3:**
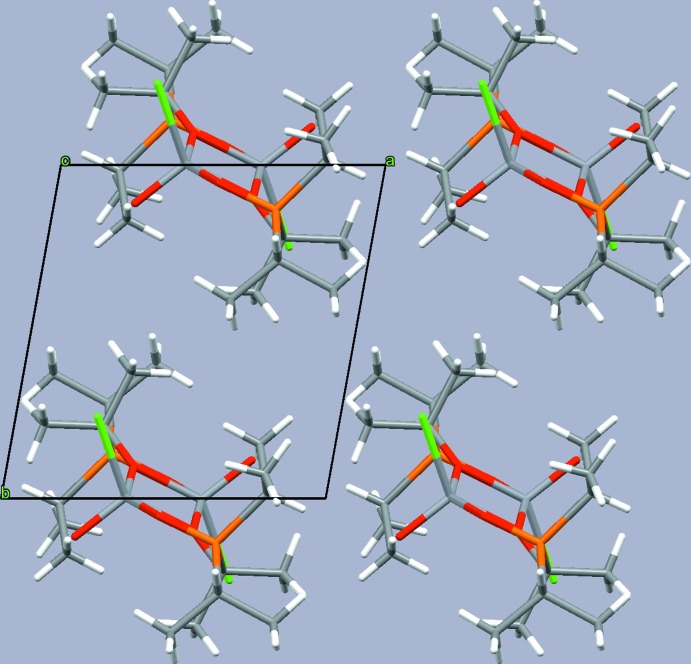
A projection of the crystal structure along [001] showing the stacking of mol­ecules of (1) in this direction.

**Figure 4:**
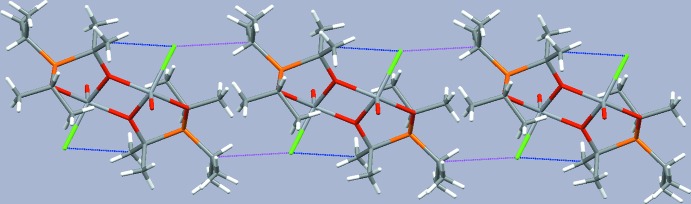
A hydrogen-bonded chain of mol­ecules extending parallel to [001]. Intra­molecular C—H⋯Cl contacts are given as blue dotted lines and inter­molecular C—H⋯Cl contacts as red dotted lines.

In the crystal, the mol­ecules are stacked into rows along [001] (Fig. 3 ▸). An intra­molecular C—H⋯Cl contact [3.425 (3) Å] involving one methyl H atom of the isopropyl moiety is present. A similar inter­molecular contact [3.578 (3) Å] between the Cl atom of one and the secondary H atom of the isopropyl moiety of an adjacent mol­ecule leads to the formation of hydrogen-bonded chains along the stacking direction (Fig. 4 ▸). Numerical details of these inter­actions are given in Table 1 ▸.

## Database survey   

A search in the Cambridge Structural Database (Groom *et al.*, 2016 ▸) for structures of compounds containing the V_2_O_2_ core and vanadium atoms additionally bonded to one Cl atom and double-bonded to one vanadyl O atom revealed 22 entries. In all these structures the coordination environment of the vanadium atoms is similar to that of the title structure.

## Synthesis and crystallization   

General. All manipulations were performed under an inert atmosphere of argon by using Schlenk techniques or in a MBraun inert-gas glovebox. The solvents were purified according to standard procedures. VCl_3_(THF)_3_ was purchased from Sigma–Aldrich and used without further purification. The synthesis of the PCP ligand employed was described in detail by Murugesan *et al.* (2014 ▸).

The oxido-vanadium complex (1) was formed during an attempt to synthesize a V^III^ PCP complex (Fig. 1 ▸). VCl_3_(THF)_3_ (75 mg, 0.20 mmol) and the PCP ligand (85 mg, 0.22 mmol) were stirred in 7 ml THF for 30 min and cooled to 195 K. Upon addition of 0.22 mmol *n*-BuLi (2.5 *M* solution in *n*-hexa­ne), the mixture was allowed to reach room temperature and was stirred for another two h. The colour changed from orange to violet. After evaporation of the solvent, the remaining solids were redissolved in 5 ml acetone and filtrated over celite. The clear violet solution was layered with 10 ml diethyl ether and was left to stand for two days. Pale violet crystals, mostly with a needle-like form, suitable for X-ray analysis were isolated. IR spectrum (Perkin–Elmer 400 FIR FTIR spectrometer, equipped with a Pike Technologies GladiATR using a diamond crystal plate): ν(V=O) 996 cm^−1^ (for the full spectrum see Supporting information).

## Refinement   

Crystal data, data collection and structure refinement details are summarized in Table 2 ▸. All H atoms were placed in calculated positions and were refined in the riding-atom approximation, with C—H = 0.96 Å and *U*_iso_(H) = 1.2*U*_eq_(C).

## Supplementary Material

Crystal structure: contains datablock(s) New_Global_Publ_Block, I. DOI: 10.1107/S2056989016007362/hb7583sup1.cif

Structure factors: contains datablock(s) I. DOI: 10.1107/S2056989016007362/hb7583Isup2.hkl

Supporting information file. DOI: 10.1107/S2056989016007362/hb7583sup3.pdf

CCDC reference: 1477727

Additional supporting information:  crystallographic information; 3D view; checkCIF report

## Figures and Tables

**Table 1:** Hydrogen-bond geometry (Å, °)

*D*—H⋯*A*	*D*—H	H⋯*A*	*D*⋯*A*	*D*—H⋯*A*
C3—H2C3⋯Cl1^i^	0.96	2.68	3.425 (3)	135
C4—H1C4⋯Cl1^ii^	0.96	2.77	3.578 (3)	142

Symmetry codes: (i) 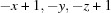; (ii) 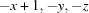.

**Table 2:** Experimental details

Crystal data	
Chemical formula	[V_2_(C_9_H_20_O_2_P)_2_Cl_2_O_2_]
*M*_r_	587.2
Crystal system, space group	Triclinic, *P* 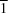
Temperature (K)	100
*a*, *b*, *c* (Å)	8.0592 (17), 8.611 (2), 10.170 (2)
α, β, γ (°)	104.148 (7), 96.778 (6), 98.132 (6)
*V* (Å^3^)	668.9 (3)
*Z*	1
Radiation type	Mo *K*α
μ (mm^−1^)	1.05
Crystal size (mm)	0.38 × 0.18 × 0.01

Data collection	
Diffractometer	Bruker Kappa APEXII CCD
Absorption correction	Multi-scan (*SADABS*; Bruker, 2015 ▸)
*T*_min_, *T*_max_	0.80, 0.99
No. of measured, independent and observed [*I* > 3σ(*I*)] reflections	13875, 3233, 2231
*R*_int_	0.053
(sin θ/λ)_max_ (Å^−1^)	0.661

Refinement	
*R*[*F* > 3σ(*F*)], *wR*(*F*), *S*	0.040, 0.044, 1.53
No. of reflections	3233
No. of parameters	136
H-atom treatment	H-atom parameters constrained
Δρ_max_, Δρ_min_ (e Å^−3^)	0.59, −0.31

Computer programs: *APEX2* and *SAINT-Plus* (Bruker, 2015 ▸), *SUPERFLIP* (Palatinus & Chapuis, 2007 ▸), *JANA2006* (Petříček *et al.*, 2014 ▸), *Mercury* (Macrae *et al.*, 2006 ▸) and *publCIF* (Westrip, 2010 ▸).

## supplementary crystallographic information

### Crystal data

**Table:**

[V_2_(C_9_H_20_O_2_P)_2_Cl_2_O_2_]	*Z* = 1
*M**_r_* = 587.2	*F*(000) = 306
Triclinic, *P*1	*D*_x_ = 1.458 Mg m^−^^3^
Hall symbol: -P 1	Mo *K*α radiation, λ = 0.71073 Å
*a* = 8.0592 (17) Å	Cell parameters from 4741 reflections
*b* = 8.611 (2) Å	θ = 2.5–25.5°
*c* = 10.170 (2) Å	µ = 1.05 mm^−^^1^
α = 104.148 (7)°	*T* = 100 K
β = 96.778 (6)°	Plate, translucent pale violet
γ = 98.132 (6)°	0.38 × 0.18 × 0.01 mm
*V* = 668.9 (3) Å^3^	

### Data collection

**Table:**

Bruker Kappa APEXII CCD diffractometer	3233 independent reflections
Radiation source: X-ray tube	2231 reflections with *I* > 3σ(*I*)
Graphite monochromator	*R*_int_ = 0.053
ω– and φ–scans	θ_max_ = 28.0°, θ_min_ = 2.1°
Absorption correction: multi-scan (*SADABS*; Bruker, 2015)	*h* = −10→10
*T*_min_ = 0.80, *T*_max_ = 0.99	*k* = −11→11
13875 measured reflections	*l* = −13→13

### Refinement

**Table:**

Refinement on *F*	80 constraints
*R*[*F* > 3σ(*F*)] = 0.040	H-atom parameters constrained
*wR*(*F*) = 0.044	Weighting scheme based on measured s.u.'s *w* = 1/(σ^2^(*F*) + 0.0001*F*^2^)
*S* = 1.53	(Δ/σ)_max_ = 0.009
3233 reflections	Δρ_max_ = 0.59 e Å^−^^3^
136 parameters	Δρ_min_ = −0.31 e Å^−^^3^
0 restraints	

### Fractional atomic coordinates and isotropic or equivalent isotropic displacement parameters (Å^2^)

**Table:**

	*x*	*y*	*z*	*U*_iso_*/*U*_eq_	
V1	0.38304 (5)	−0.00427 (6)	0.36112 (5)	0.01926 (17)	
Cl1	0.25262 (9)	−0.24490 (9)	0.20308 (7)	0.0341 (3)	
P1	0.68528 (8)	0.13495 (8)	0.24674 (7)	0.0173 (2)	
O1	0.49776 (19)	0.0539 (2)	0.21289 (17)	0.0192 (6)	
O2	0.60943 (19)	0.0969 (2)	0.47621 (17)	0.0174 (6)	
O3	0.2494 (2)	0.1146 (2)	0.38388 (19)	0.0281 (7)	
C1	0.7236 (3)	0.2170 (3)	0.4368 (2)	0.0173 (9)	
C2	0.6686 (3)	0.3802 (3)	0.4827 (3)	0.0256 (10)	
C3	0.9074 (3)	0.2240 (3)	0.4969 (3)	0.0213 (9)	
C4	0.7223 (3)	0.2850 (3)	0.1523 (3)	0.0260 (10)	
C5	0.5959 (4)	0.4029 (3)	0.1606 (3)	0.0369 (12)	
C6	0.9071 (4)	0.3734 (3)	0.1821 (3)	0.0342 (11)	
C7	0.8172 (3)	−0.0168 (3)	0.1968 (3)	0.0215 (9)	
C8	0.7671 (3)	−0.1627 (3)	0.2529 (3)	0.0289 (11)	
C9	0.7981 (4)	−0.0735 (4)	0.0400 (3)	0.0337 (11)	
H1c2	0.745608	0.462229	0.460631	0.0307*	
H2c2	0.556075	0.374592	0.436426	0.0307*	
H3c2	0.669143	0.407097	0.580109	0.0307*	
H1c3	0.981032	0.288554	0.455184	0.0256*	
H2c3	0.924596	0.272033	0.594195	0.0256*	
H3c3	0.932653	0.115831	0.479036	0.0256*	
H1c4	0.700955	0.222399	0.058035	0.0312*	
H1c5	0.483119	0.343833	0.152296	0.0443*	
H2c5	0.623875	0.483795	0.247252	0.0443*	
H3c5	0.601381	0.454885	0.0874	0.0443*	
H1c6	0.981535	0.294962	0.173736	0.041*	
H2c6	0.924906	0.436542	0.117545	0.041*	
H3c6	0.930691	0.444049	0.273627	0.041*	
H1c7	0.933264	0.031353	0.234338	0.0257*	
H1c8	0.771966	−0.126298	0.350545	0.0347*	
H2c8	0.653646	−0.215951	0.210932	0.0347*	
H3c8	0.844026	−0.237513	0.232403	0.0347*	
H1c9	0.851769	0.012298	0.006097	0.0404*	
H2c9	0.850708	−0.167368	0.014034	0.0404*	
H3c9	0.679713	−0.1011	0.001481	0.0404*	

### Atomic displacement parameters (Å^2^)

**Table:**

	*U*^11^	*U*^22^	*U*^33^	*U*^12^	*U*^13^	*U*^23^
V1	0.0077 (2)	0.0305 (3)	0.0189 (2)	0.00292 (19)	−0.00264 (18)	0.0079 (2)
Cl1	0.0313 (4)	0.0409 (5)	0.0202 (4)	−0.0142 (3)	−0.0024 (3)	0.0041 (3)
P1	0.0116 (3)	0.0196 (4)	0.0192 (4)	0.0018 (3)	−0.0006 (3)	0.0044 (3)
O1	0.0098 (9)	0.0280 (11)	0.0191 (9)	0.0024 (8)	−0.0036 (7)	0.0082 (8)
O2	0.0074 (8)	0.0242 (10)	0.0193 (9)	−0.0004 (7)	−0.0018 (7)	0.0070 (8)
O3	0.0147 (10)	0.0439 (13)	0.0314 (11)	0.0116 (9)	0.0038 (8)	0.0169 (10)
C1	0.0133 (13)	0.0208 (15)	0.0166 (13)	0.0028 (11)	−0.0006 (10)	0.0045 (11)
C2	0.0190 (14)	0.0269 (16)	0.0287 (16)	0.0041 (12)	0.0021 (12)	0.0039 (13)
C3	0.0123 (13)	0.0274 (16)	0.0206 (14)	−0.0008 (11)	−0.0014 (11)	0.0040 (12)
C4	0.0270 (15)	0.0247 (16)	0.0244 (15)	−0.0028 (13)	0.0001 (12)	0.0093 (13)
C5	0.0474 (19)	0.0229 (17)	0.0393 (19)	0.0034 (15)	−0.0047 (16)	0.0134 (14)
C6	0.0380 (17)	0.0282 (17)	0.0328 (18)	−0.0055 (14)	0.0060 (15)	0.0078 (15)
C7	0.0118 (13)	0.0256 (16)	0.0228 (14)	0.0054 (11)	−0.0007 (11)	−0.0008 (12)
C8	0.0236 (15)	0.0246 (16)	0.0357 (17)	0.0110 (13)	−0.0030 (13)	0.0023 (14)
C9	0.0290 (17)	0.0390 (19)	0.0289 (16)	0.0106 (15)	0.0038 (14)	−0.0013 (14)

### Geometric parameters (Å, º)

**Table:**

V1—Cl1	2.3105 (13)	C4—C5	1.532 (4)
V1—O1	1.986 (2)	C4—C6	1.533 (4)
V1—O2	2.0014 (17)	C4—H1c4	0.96
V1—O2^i^	2.003 (2)	C5—H1c5	0.96
V1—O3	1.586 (2)	C5—H2c5	0.96
P1—O1	1.5333 (17)	C5—H3c5	0.96
P1—C1	1.861 (3)	C6—H1c6	0.96
P1—C4	1.802 (3)	C6—H2c6	0.96
P1—C7	1.814 (3)	C6—H3c6	0.96
O2—C1	1.448 (3)	C7—C8	1.525 (4)
C1—C2	1.514 (4)	C7—C9	1.532 (4)
C1—C3	1.522 (3)	C7—H1c7	0.96
C2—H1c2	0.96	C8—H1c8	0.96
C2—H2c2	0.96	C8—H2c8	0.96
C2—H3c2	0.96	C8—H3c8	0.96
C3—H1c3	0.96	C9—H1c9	0.96
C3—H2c3	0.96	C9—H2c9	0.96
C3—H3c3	0.96	C9—H3c9	0.96
			
Cl1—V1—O1	87.75 (5)	H2c3—C3—H3c3	109.47
Cl1—V1—O2	139.45 (6)	P1—C4—C5	114.9 (2)
Cl1—V1—O2^i^	95.33 (5)	P1—C4—C6	112.9 (2)
Cl1—V1—O3	108.79 (6)	P1—C4—H1c4	103.96
O1—V1—O2	82.92 (7)	C5—C4—C6	112.4 (2)
O1—V1—O2^i^	148.82 (7)	C5—C4—H1c4	104.59
O1—V1—O3	103.91 (10)	C6—C4—H1c4	107.07
O2—V1—O2^i^	74.64 (7)	C4—C5—H1c5	109.47
O2—V1—O3	111.77 (8)	C4—C5—H2c5	109.47
O2^i^—V1—O3	104.38 (9)	C4—C5—H3c5	109.47
O1—P1—C1	104.13 (10)	H1c5—C5—H2c5	109.47
O1—P1—C4	109.68 (11)	H1c5—C5—H3c5	109.47
O1—P1—C7	109.71 (10)	H2c5—C5—H3c5	109.47
C1—P1—C4	114.97 (12)	C4—C6—H1c6	109.47
C1—P1—C7	109.83 (12)	C4—C6—H2c6	109.47
C4—P1—C7	108.40 (13)	C4—C6—H3c6	109.47
V1—O1—P1	118.99 (10)	H1c6—C6—H2c6	109.47
V1—O2—V1^i^	105.36 (8)	H1c6—C6—H3c6	109.47
V1—O2—C1	120.68 (14)	H2c6—C6—H3c6	109.47
V1^i^—O2—C1	133.87 (13)	P1—C7—C8	110.47 (19)
P1—C1—O2	100.94 (13)	P1—C7—C9	110.0 (2)
P1—C1—C2	112.56 (19)	P1—C7—H1c7	108.43
P1—C1—C3	111.43 (17)	C8—C7—C9	109.4 (2)
O2—C1—C2	108.3 (2)	C8—C7—H1c7	109.02
O2—C1—C3	111.6 (2)	C9—C7—H1c7	109.51
C2—C1—C3	111.50 (18)	C7—C8—H1c8	109.47
C1—C2—H1c2	109.47	C7—C8—H2c8	109.47
C1—C2—H2c2	109.47	C7—C8—H3c8	109.47
C1—C2—H3c2	109.47	H1c8—C8—H2c8	109.47
H1c2—C2—H2c2	109.47	H1c8—C8—H3c8	109.47
H1c2—C2—H3c2	109.47	H2c8—C8—H3c8	109.47
H2c2—C2—H3c2	109.47	C7—C9—H1c9	109.47
C1—C3—H1c3	109.47	C7—C9—H2c9	109.47
C1—C3—H2c3	109.47	C7—C9—H3c9	109.47
C1—C3—H3c3	109.47	H1c9—C9—H2c9	109.47
H1c3—C3—H2c3	109.47	H1c9—C9—H3c9	109.47
H1c3—C3—H3c3	109.47	H2c9—C9—H3c9	109.47

Symmetry code: (i) −*x*+1, −*y*, −*z*+1.

### Hydrogen-bond geometry (Å, º)

**Table:**

*D*—H···*A*	*D*—H	H···*A*	*D*···*A*	*D*—H···*A*
C3—H2C3···Cl1^i^	0.96	2.68	3.425 (3)	135
C4—H1C4···Cl1^ii^	0.96	2.77	3.578 (3)	142

Symmetry codes: (i) −*x*+1, −*y*, −*z*+1; (ii) −*x*+1, −*y*, −*z*.
