# Supplementary material for: Crystal structure of bis­[μ-2-(diiso­propyl­phosphor­yl)propan-2-olato-κ3 O 1,O 2:O 1]bis­[chlorido­oxidovanadium(IV)]
Source: Acta Crystallogr E Crystallogr Commun. 2016 May 6;72(Pt 6):785–8. doi: 10.1107/S2056989016007362 (PMC4908574; doi:10.1107/S2056989016007362)
Supplement: Supplementary file 3 [file e-72-00785-sup3.pdf]

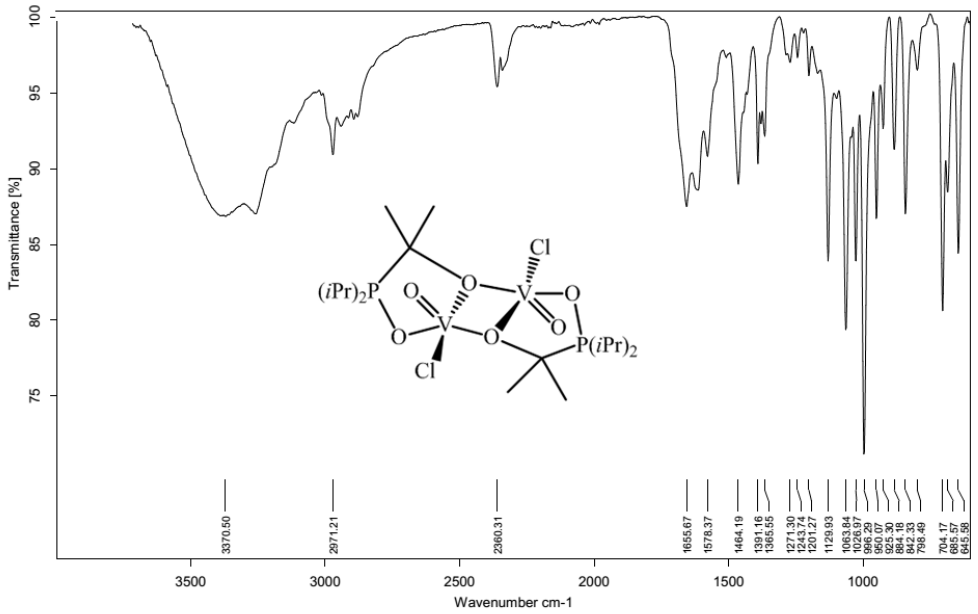


**Supplementary Figure.** IR spectrum of **1**, showing an intensive stretching frequency $\nu_{VO}$ at 996 $cm^{-1}$.
